# The NHS Health Check programme: a survey of programme delivery in England before and after the Covid-19 pandemic response

**DOI:** 10.3310/nihropenres.13436.2

**Published:** 2023-08-25

**Authors:** Erica Wirrmann Gadsby, Janet Krska, Claire Duddy, Vivienne Hibberd, Geoff Wong

**Affiliations:** 1 Faculty of Health Sciences and Sport, University of Stirling, Stirling, Scotland, FK9 4LA, UK; 2 Centre for Health Services Studies, University of Kent, Canterbury, England, CT2 7NX, UK; 3 Medway School of Pharmacy, Universities of Greenwich and Kent, Medway, ME4 4TB, UK; 4 Nuffield Department of Primary Care Health Sciences, University of Oxford, Oxford, OX2 6GG, UK; 5 Public Involvement in Pharmacy Studies Group, Medway School of Pharmacy, Medway, ME4 4TB, UK

**Keywords:** local government, public health, cardiovascular disease, prevention, health check

## Abstract

**Background:**

This study investigated NHS Health Check programme delivery before and after the Covid-19 pandemic response, with a focus on support services and referral methods available to Health Check attendees. The NHS Health Check is an important part of England’s Cardiovascular Disease (CVD) prevention programme.

**Methods:**

Public health commissioners from all 151 local authorities responsible for commissioning the NHS Health Check programme were surveyed in 2021, using an online questionnaire to capture detail about programme delivery, changes in delivery because of the pandemic response, and monitoring of programme outcomes. Four-point rating scales were used to obtain level of confidence in capacity, accessibility and usage of follow-on support services for Health Check attendees. A typology of programme delivery was developed, and associations between delivery categories and a range of relevant variables were assessed using one-way analysis of variance.

**Results:**

Sixty-eight responses were received on behalf of 74 (of 151) local authorities (49%), across all geographical regions. Our findings suggest a basic typology of delivery, though with considerable variation in who is providing the Checks, where and how, and with continued changes prompted by the Covid-19 pandemic. Support for risk management is highly varied with notable gaps in some areas. Local authorities using a model of delivery that includes community venues tended to have a higher number of services to support behaviour change following the Check, and greater confidence in the accessibility and usage of these services. A minority of local authorities gather data on referrals for Health Check attendees, or on outcomes of referrals.

**Conclusions:**

The Covid-19 pandemic has prompted continued changes in delivery, which are likely to influence patient experience and outcomes; these need careful evaluation. The programme’s delivery and commissioners’ intentions to follow through risk communication with appropriate support is challenged by the complexity of the commissioning landscape.

## Plain English summary

The NHS Health Check programme is for adults in England aged between 40 and 74. It aims to help people to reduce their risk of some major conditions such as heart disease and stroke. The Check involves taking measurements and calculating a 'risk score'. People considered 'at risk' are then advised on how to reduce this. Professionals can refer people to services that will help, like weight management services. Different professionals might provide the Checks in various settings. This depends on choices made by local councils. How and where they are provided, and how well they work, differs across England.

Our study helped us learn more about this programme. We focused on what happens after people are told their risk score. There are 151 local councils that make decisions about how to deliver the Health Check programme in their area. We contacted these councils in 2021 to ask them questions in an online questionnaire. These questions were about programme delivery, including during the Covid-19 pandemic. And about whether and how they track programme outcomes. We also asked about the follow-on support services that are available for people. Using this information, we developed broad categories of programme delivery.

About half of all local councils responded. Our findings show lots of variation in who is providing the Checks, where and how. The Covid-19 pandemic prompted even more changes. However, we were able to suggest three broad categories of delivery based on our findings. Support for people to manage their disease risk varies, with notable gaps in some areas. Most local councils gather very little information on what happens to people who attend the Health Checks. We need to know more about how different ways of delivering Health Checks affects patient experience and outcomes.

## Introduction

The National Health Service (NHS) five-yearly Health Check is an important part of England’s cardiovascular disease (CVD) prevention programme ^1^; it features in the NHS Long Term Plan ^2^ and strategy for addressing health inequalities ^3^. In theory, eligible adults (aged 40–74, without a pre-existing CVD-related health condition) are invited to undergo a Check in which a range of behavioural and physiological risk factors are assessed. Depending on their risk score, people are then given personalised advice on how to reduce their risk and referred for further assessment (e.g., for diabetes) and/or intervention (e.g., medication prescription, weight management, smoking cessation) as appropriate. The achievement of longer-term health outcomes depends on both effective delivery of the programme, and Health Check attendees subsequently taking action to manage or reduce their CVD risk.

Whilst there are no set targets for uptake of NHS Health Checks, the estimated uptake rate was originally modelled at 75% ^4^. An evaluation of uptake during a five year cycle (2012 to 2017) found that on average, 52.6% took up the offer, with uptake within different local areas ranging from 25% to 85% ^5^. There has been considerable attention paid to the lower-than-anticipated uptake of Health Checks ^6, 7^, and on the large variation in delivery of this national programme ^8, 9^. However, there has been very little attention paid to post-Check management of those identified as having modifiable risk factors. A limited number of studies have demonstrated mixed results on post-Health Check improvements in relevant risk factors ^9^, and there is a notable lack of understanding of what happens in practice, after a person’s risk has been assessed.

In England, there are 151 upper-tier and unitary local government councils/authorities, which have a statutory responsibility for improving the health of their local population and for certain public health services as defined by the Health and Social Care Act 2012 ^10^. The Act specifies the duties of local authorities (LAs) to deliver the NHS Health Check programme, and a portion of the public health grant paid to local authorities from the Department of Health and Social Care budget is designated for the commissioning of NHS Health Checks. Overseen by the Office for Health Improvement and Disparities (OHID), they have considerable flexibility in who to commission and how to deliver the programme. In fulfilling their public health duties, LAs work closely with NHS bodies which, at a local level, have undergone several recent rounds of reform ^11^. Consequently, the local picture of public health service commissioning and delivery is complex, and Checks are delivered by a range of providers in different settings.

Local Authorities Regulations (2013) stipulate the measures that should be recorded (and forwarded to the individual’s GP) as part of a Check: age, gender, smoking status, family history of CHD, ethnicity, body mass index, cholesterol level, blood pressure, physical activity level, alcohol use disorders identification test score, and cardiovascular risk score ^12^. NHS Health Check programme standards introduced in 2014 and most recently updated in 2020, set out minimum (not mandatory) standards necessary to deliver a safe and effective Check ^13^. Commissioners and providers of the programme are also supported with ‘best practice guidance’ ^14^. Local authorities have a statutory duty to provide data for each financial quarter on the number of NHS Health Checks offered, and the number of NHS Health Checks received. This data is quality assured and published as official statistics. Two national surveys have previously highlighted the considerable variation in delivery practice across England ^15, 16^. Both focused almost exclusively on issues to do with prioritisation, invitation, and delivery of the initial steps of the Health Check pathway (involving risk assessment and communication). Only the 2014 survey included a question on provision of support for lifestyle change.

Our research aimed to fill the gap in understanding of what happens after the risk assessment. The project involved a survey of LA commissioners, a realist review of the literature ^17^, and stakeholder engagement with professionals (policy makers, commissioners, trainers and providers of Health Checks) and members of the public. The cross-sectional survey was to investigate any variation in the support services and referral methods available to Health Check attendees, as well as gain more detailed information about Health Check provision. Given the project’s timing (2021), it also examined changes in delivery caused by the Covid-19 pandemic. In general, programme delivery was paused in April 2020 for a full year, with service resumption depending on local safety arrangements and the need to prioritise the Covid-19 vaccination programme. Even in May 2022, according to the patient-facing NHS Health Check website, delivery remained paused in some areas.

This paper reports the survey findings to describe how Health Checks are delivered across England, particularly in relation to follow-on services supporting risk management, and to determine how the Covid-19 pandemic has changed delivery. It develops a typology of NHS Health Check programme delivery and examines associations between delivery of the Health Check and other relevant aspects, including support services available and NHS Health Check programme performance.

## Methods

### Patient and Public Involvement

Prior to project funding, we involved members of the public and charities in the drafting of the protocol. We sought feedback on the importance of the proposed study and on what we should focus on from members of two established public engagement in research groups (Public Involvement in Pharmacy Studies at Medway School of Pharmacy and Opening Doors to Research at the Centre for Health Services Studies), and from representatives from several charities with an interest in NHS Health Checks. Feedback highlighted the importance of investigating variation in experience and quality of the NHS Health Check by region and by professional groups delivering them, and outcomes related to NHS Health Check attendance, including changes in lifestyle. This feedback informed our research questions and study design and highlighted the importance of a survey alongside our realist review of the literature.

Throughout the project, our strategy for patient and public involvement (PPI) was informed by our PPI lead and co-author (VH). VH is an experienced PPI contributor who brought her valuable perspective as a member of the public, and skills in group facilitation. Our PPI group involved ten members of the public from six different English regions, all of whom were eligible to receive the NHS Health Check. Members were purposively selected to be as diverse as possible in relation to gender, age, ethnicity and geographical location, with the aim of capturing a range of different perspectives from individual members of the public. The group was consulted via regular online meetings throughout the project, using Zoom (Version 5, Zoom Video Communications, Inc.). They provided feedback on our draft survey questions, discussed our emerging findings, helped to shape our analysis, helped to develop practical recommendations for NHS Health Check delivery, and informed our dissemination strategies. 

A separate professional stakeholder group also provided us with content expertise and a range of perspectives throughout the project, via regular online meetings using Microsoft Teams (Version 1.0, Microsoft Corporation). For this group, we recruited (via our project team’s existing networks and snowballing from these) 14 people across the following categories: LA commissioners, NHS Health Check providers, NHS Health Check trainers, and representatives from relevant health charities. In addition, we maintained close contact with Public Health England’s (latterly the Office for Health Improvement and Disparities) CVD Prevention Programme Lead.

The online survey (see extended data: supplementary file ^18^) was developed in collaboration with our professional stakeholder group, using Jisc Online Surveys, to complement data gathered in the previous national survey of 2020, and also to capture a) detail about the delivery of the programme following the risk assessment, and b) any changes in delivery because of the pandemic response. It asked how the programme outcomes were monitored and whether LAs had commissioned, conducted or been part of any assessments of the programme in the previous five years. It included a mix of closed- and open-ended questions (19 in total), the latter to enable respondents to explain their responses and add any further relevant information about commissioning and delivery (illustrative extracts of these are presented in the Results). Four-point rating scales were used to obtain level of confidence in capacity (considering e.g., eligibility criteria, waiting times), accessibility (considering e.g., opening times, location, cost to users), and usage of support services (range from 1 ‘not confident at all’ to 4 ‘very confident’). Designed to be completed by a lead person for CVD prevention or Health Check commissioning in each LA, the survey was piloted within seven LAs; three completed it fully and provided positive feedback, with one subsequent minor change made prior to launch.

Ethics approval was granted by the University of Kent SRC Ethical Review Panel (Division of Law, Society and Social Justice) (SRCEA id 0367).

The survey (with direct link and password) was disseminated to key individuals in all 151 LAs in England with responsibility for commissioning the NHS Health Check programme. Public Health England (PHE) (at the time responsible for overseeing the programme at national level) sent the survey on our behalf, using the same distribution methods as for their own previous surveys, via regional Health Check leads and the programme’s Local Implementor National Forum. It was also publicised via the established NHS Health Check webinar series. It opened 17 ^th^ May and closed 18 ^th^ July 2021, with two reminder emails sent by PHE, and a third targeted reminder sent to Health Check leads in regions where the response rate was below 35%. The questionnaire was preceded by an information page, a privacy notice and a consent form. Active consent was required to proceed with the form. Individuals completing the survey were invited to provide their name and email address if they wished, to allow us to send the findings of our research. Names and contact information were separated from the data file and stored separately from all other information.

Survey responses were downloaded into Microsoft Excel v2304 (RRID:SCR_016137) and IBM SPSS Statistics v27 (RRID:SCR_019096). Where individuals responded for more than one LA (where LA public health teams are merged), unique identifiers were given to both respondents and LAs. Single responses provided on behalf of multiple LAs were copied exactly. Open-ended question responses were used to clarify or amend responses where relevant.

### Data analysis

Simple descriptive statistics were used to analyse quantitative responses, which were enhanced and illustrated by qualitative responses, where the open comments sometimes provided clarification or further detail. One response was amended, where the respondent had not ticked ‘pharmacist’ as a provider, but had told us in a free text comment that pharmacists were used to deliver the Checks. A typology of Health Check delivery was derived from the data, in consultation with our stakeholder group (see Results).

The research team and stakeholder groups were interested in whether delivery method was associated with support service availability, accessibility and use, and whether commissioners with more complex delivery methods also used additional factors to prioritise Health Check invitations and/or engaged in more monitoring of their providers. Therefore, we assessed associations between category of delivery and: total number of commissioned support services reported, total number of referral processes reported, average reported confidence in capacity, accessibility and usage of support services, total number of methods used to prioritise invitations pre- and post-Covid-19 pause, and total monitoring and evaluation reported.

Our emerging review findings and discussions with our professional stakeholder group pointed to additional key variables understood to be of significance to Health Check programme delivery. For each of these variables, statistics were obtained from publicly available data, and associations assessed between these and delivery category. The variables used were: geographic region; estimated population for 2019; Public Health budget per head, 2019/20; Indices of Multiple Deprivation (IMD), 2019; and proportion of eligible people receiving a Check between 2015/16 and 2019/20 https://www.healthcheck.nhs.uk/. Rurality was an additional factor perceived likely to influence Health Check delivery but was not possible to include given the way it is classified at district, rather than county council level.

One-way analysis of variance was used to assess associations. Relationships between variables were assessed using Spearman’s correlation coefficient.

## Results

There were four LAs for which there were two identical responses. Removal of these duplicates left 68 responses (R01–R68), reporting on behalf of 74 LAs (LA01–LA74). Whilst the overall response rate was 49%, the proportion of responses varied across PHE geographic regions, ranging from 29% of LAs in West Midlands region to 72% of LAs in the South East ( Table 1).

**Table 1.** Survey respondents by region.

Geographic region	Number of LAs	Number of Responding LAs	% of Responding LAs
East Midlands	9	5	56%
East of England	12	8	67%
London	33	18	55%
North East	12	4	33%
North West	23	9	39%
South East	18	13	72%
South West	15	6	40%
West Midlands	14	4	29%
Yorks & Humber	15	7	47%
Total	151	74	49%

**LAs: local authorities**

### Typology of NHS Health Check delivery

A typology of delivery was developed through an iterative process, looking across the data to compare and contrast delivery locations, modes and staff involved (these aspects are presented in more detail in the sub-sections below). The professional stakeholder group were consulted regarding various iterations of the typology. The 74 LAs represented in our sample were categorised as:

- General practice delivery (24; 32%): delivered in the general practice setting only, by one or more of the following staff: GP, nurse, health care assistant, health trainer, and pharmacist. No remote methods used for delivering any aspect of the Check.
- Blended delivery (21; 28%): seven LAs delivered in GP practices and pharmacies, with or without some use of remote methods post Covid-19 pause. All others delivered in GP practices only, but also used remote methods to deliver part of the Check. They were delivered by one or more of the following staff: GP, nurse, health care assistant, pharmacist, pharmacy assistant, and paramedic.
- **Blended with outreach delivery** (29; 39%): all LAs except one delivered the Check in GP practices. In addition, they all delivered the Check in at least one other venue, one of which was a community venue other than a pharmacy. Eighteen LAs in this category used no remote methods. Eleven used some form of remote methods.

**Figure 1.**
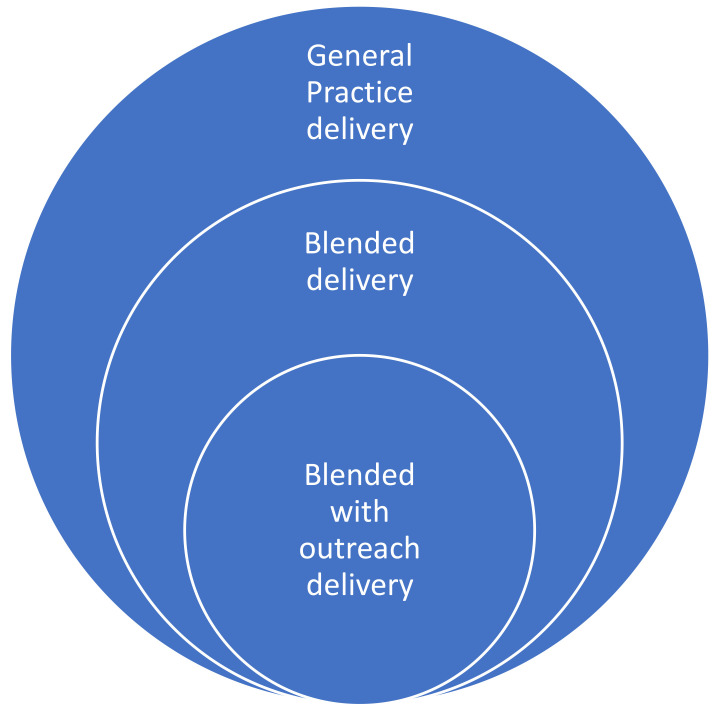
Typology of NHS Health Check delivery.

This typology is depicted diagrammatically in Figure 1. 

### Location and mode of Health Check delivery

In 64/74 LAs, delivery of Checks had resumed since the Covid-19 pause. General practice was the most common setting for face-to-face delivery both pre- (73/74) and post-Covid pause (64/64). Other commonly used venues pre-Covid were community settings such as workplaces, community centres and places of worship (n = 30; 41%) and pharmacies (n = 16; 22%); however, these had reduced after the pause in delivery to 15 and 9 respectively. The use of alternative (remote) methods of delivery increased, mostly via the telephone (two pre- and 26 post-Covid pause) and video (one pre- and 11 post-Covid) and the range of venues expanded to include a Covid vaccination centre. Several LAs used multiple remote methods, including on-line self-completion of the Health Check assessment. Two had used a remote digital check pre-Covid; one discontinued this post-Covid pause, while the other increased its availability.

To provide partially remote Health Checks, 11 LAs required the patient to attend the practice for blood tests; six others used data on file, providing they were sufficiently recent. In two LAs, a drive-through blood testing service was used. The future potential use of remote methods combined with face-to-face testing was regarded positively by most respondents (n = 54; 75%). Several described how they were operationalising these, for example:

We are testing the feasibility and acceptability of remote blood testing using a kiosk in community setting and then linking back to an online questionnaire tool. This system would be for lower risk patients predominantly. (R61)

Online completion of a lifestyle questionnaire prior to a face-to-face appointment was variously seen as a way of reducing the time taken for the Check, but also offering the potential for prioritising those most able to benefit and to enable the face-to-face appointment to focus on risk communication, personal support and advice. Conversely, others (e.g., LA04, LA07, LA20) were gathering the physical measurements in a face-to-face appointment followed by a telephone consultation to discuss the results and offer interventions. Many were uncertain about what would work best in the future. Some were trialling these new methods; others were waiting for guidance on how to proceed.

### Health professionals involved in Health Check delivery

Several respondents indicated within the open comments some uncertainty about which health professionals were currently employed (in 2021/2022) to deliver the programme, due to the commissioning process. For example:

We commission GP practices to deliver it through suitably qualified, trained (and overseen) staff. It is then up to them who that actually is. (R30)

Overall, respondents indicated that the professionals most frequently involved in delivering Checks were health care assistants and/or nurses, followed by GPs, both face-to-face and remotely ( Table 2). Other providers included wellbeing advisers, health improvement practitioners, lifestyle coaches and paramedics. Seven respondents reported that the category of professional delivering the Checks had changed following the Covid pause. Delivery by leisure centre and primary care staff was reported to be more problematic. As staff were mobilised for the vaccination campaigns, more healthcare assistants were involved in Health Check delivery and fewer nurses or GPs. Some areas reported making less use of health trainers, and in some areas LA staff and paramedics were taking over delivery.

**Table 2.** Health professionals providing NHS Health Checks.

Number of professionals involved in providing NHS Health Checks		
	Face to face	Remotely
Health care assistant	61	19
Nurse	61	18
GP	48	12
Pharmacist	17	1
Pharmacy assistant	14	1
Health trainer	14	5
Other	9	3

### Prioritisation of eligible candidates

Data in Table 3 shows that many LAs did not consistently use factors such as ethnicity, deprivation or other risk factors to prioritise invitations to the Health Check. The most frequently used factor for prioritisation pre-Covid was deprivation ( Table 3). After the Covid-19 pause, more LAs used a range of factors to target individuals, including Covid risk, with some areas starting to prioritise where they hadn’t previously done so.

**Table 3.** Factors used to prioritise invitations to a Health Check before and after the Covid-19 pause.

Factors used to prioritise Health Check invitations	Number of LAs using each factor Before Covid (N = 74)	Number of LAs using each factor After Covid Pause (N = 74)
Ethnicity	22 (30%)	35 (47%)
COVID risk	-	21 (28%)
Deprivation	31 (42%)	34 (46%)
Other	28 (38%)	33 (45%)

**LAs: local authorities**

Examples cited in the ‘other’ factors category included: diagnosed mental illness, homeless, from a traveller community, inactivity, routine and manual workers, and gender/age (e.g., men over 65).

Some respondents recognised that targeting by risk was desirable but not always feasible, as illustrated by the following comment:

Smoking status, BMI above 30, Family history, ethnicity other than white, deprivation quintile 1. Practices tell us that this data is not always up to date and so it’s not easy to identify those at higher risk. (R13)

### Services to support risk reduction/management

The majority of responding authorities reported at least one service being available to support smoking cessation, alcohol and drug misuse, weight management, diabetes prevention, psychological support and social prescribing; some indicated no service, or that they didn’t know who provided the service ( Table 4). Many reported having more than one provider for some services. Five respondents reported also commissioning physical activity/exercise on referral services. Most of these services were provided through public, private or third sector commissioning; some were provided by GP or LA staff.

**Table 4.** Number of local authorities (LAs) offering support services.

Service	Number of LAs in survey sample (N=74) commissioning:					
	Don’t know who commissions	No service provider/no response	1 service provider	2 service providers	3 service providers	4 service providers
Smoking cessation	1 (1%)	2 (3%)	45 (61%)	18 (24%)	8 (11%)	
Alcohol and drug misuse	3 (4%)	0 (0%)	52 (73%)	15 (20%)	3 (4%)	1 (1%)
Weight management	5 (7%)	6 (8%)	45 (61%)	16 (22%)	2 (3%)	
Diabetes Prevention	7 (10%)	2 (3%)	60 (81%)	4 (5%)	1 (1%)	
Psychological support	20 (27%)	6 (8%)	39 (53%)	7 (10%)	1 (1%)	1 (1%)
Social prescribing	10 (14%)	2 (3%)	46 (62%)	13 (18%)	3 (4%)	
Other services			7 (10%)			

Most respondents provided ratings for their level of confidence in the capacity and accessibility of the support services available in their LAs. However, several felt unable to do so, especially for services that were not commissioned by the LA. This is reflected in the variable amount of missing data and in several comments from respondents, which highlighted the difficulty of pulling together information across a very fragmented system.

**Figure 2.**
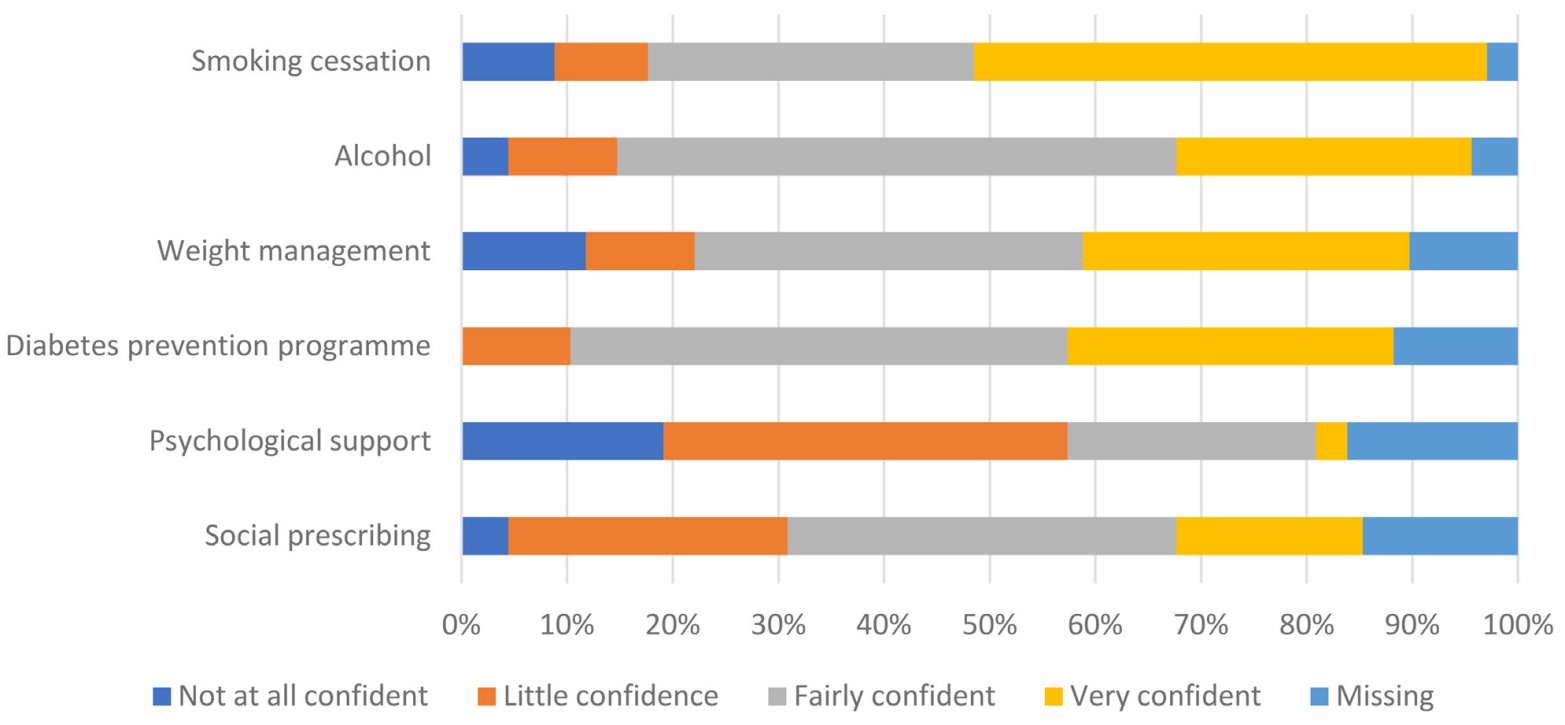
Commissioners’ confidence in the capacity of services to support Health Check patients.

Confidence in the capacity to support Health Check referred patients was mostly positive, but this varied across the different services (see Figure 2).

Several comments explained the variation in level of confidence around capacity, including reasons for poor provision, with one reporting no local services at all for weight management and stop smoking services:

If all who are eligible and ready to be referred were so, there would not be enough capacity in the system, specifically weight management and mental health alone. (R08)
  With austerity measures, the capacity in the system has become so limited that there is very little capacity available. (R13)

**Figure 3.**
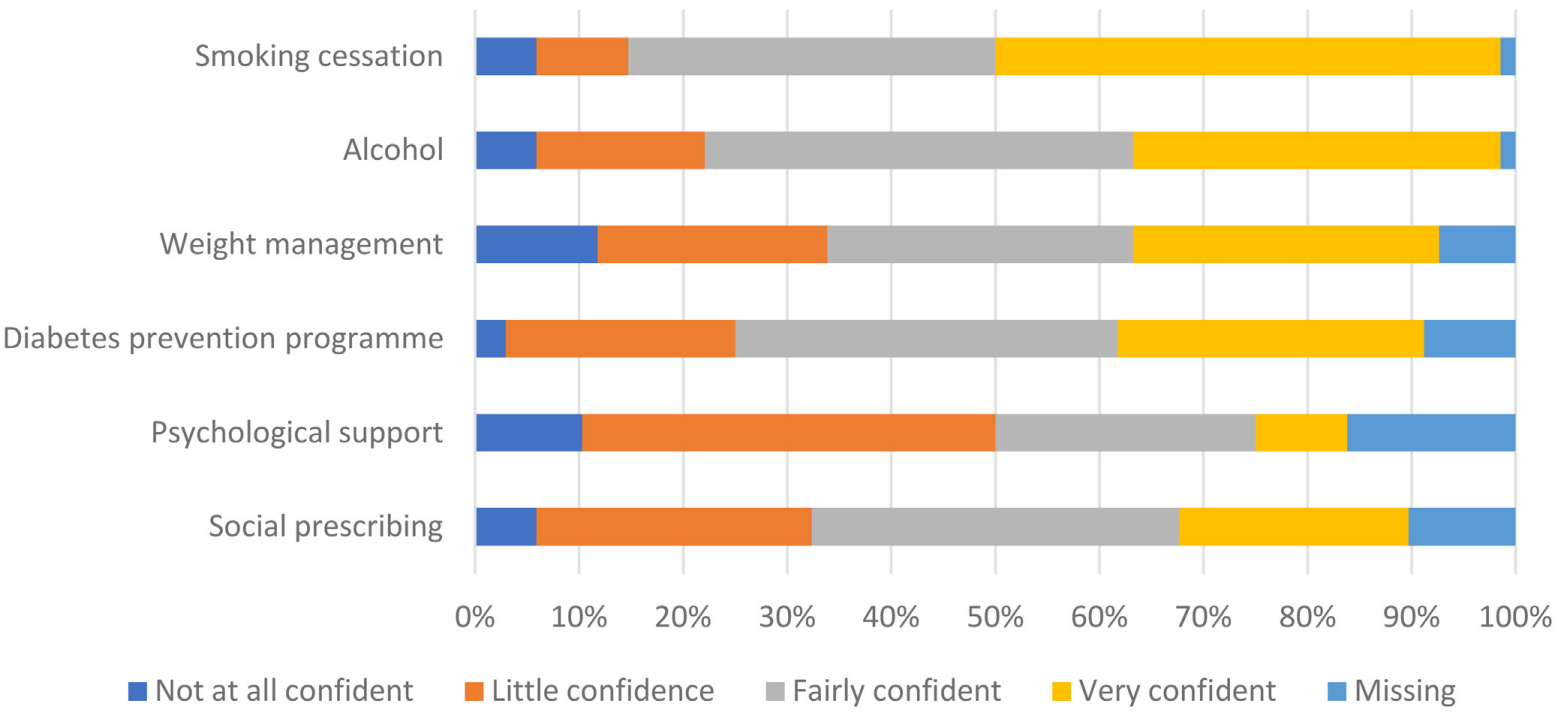
Commissioners’ confidence in the accessibility of services to support Health Check patients.

Confidence in the accessibility of the services ( Figure 3) was similar and directly correlated to confidence in the capacity (Spearman’s r = 0.752; p <0.001). Respondents were least certain about whether psychological support services had sufficient capacity and accessibility.

All respondents provided an overall confidence rating for the extent to which appropriate use was made by Health Check providers of the available support services. This was generally low, with over half of the respondents (n = 50; 68%) having relatively little/no confidence. Reasons for the lack of confidence varied, including lack of awareness among providers and lack of data on use of these services:

We do highlight support services as part of our NHS Health check training but have no way of following patients through to know how many access support successfully. (R64)
  Onward referrals from Health Checks into our own services are higher from our own team of Health Trainers, which suggests that we aren’t getting all the referrals we should be getting from other providers. (R23)

Changes in either capacity or accessibility of support services since the Covid-19 pause were reported by 40 respondents while 13 reported no change, and 15 didn’t know. Several described the impact of moving support services to online provision as affecting both accessibility and capacity both positively and negatively.

Many of these services have had to move online for part of the time at least, possibly making them more accessible for some but less accessible for others. (R50)
  Services moved onto telephone support where possible and this increased accessibility for people as they did not have to travel to attend appointments. (R66)
  F2F [face-to-face] services have moved online only which are more popular and more efficient, permitting advisers to see more clients. (R40)

In some areas, Covid-19 has prompted greater investment in support services, e.g., increased funding for and an expansion of tier-two weight management service, more social prescribing due to an increase in Primary Care Network link workers, and more funding for substance misuse services.

### Referral processes

Multiple processes were reported for enabling Health Check attendees to access follow-on services in many LAs, although some respondents did not know the referral processes in place in their area ( Table 5). As with the services themselves, awareness of the referral processes was least well known for psychological support services. Smoking cessation services had the greatest range of referral options. The number of referral processes reported was strongly related to the number of services commissioned across the LAs (Spearman’s r = 0.423; p <0.001).

**Table 5.** Number of local authorities (LAs) offering referral processes.

Service	Number of LAs reporting referral routes (N=74):					
	Client makes own appointment	Provider makes appointment	Referral via link worker	Other	Don’t know	Missing response
**Smoking cessation**	59 (80%)	41 (57%)	22 (30%)	2 (3%)	4 (5%)	3 (4%)
**Alcohol**	42 (57%)	38 (51%)	24 (32%)	1 (1%)	16 (22%)	1 (1%)
**Weight management**	37 (50%)	34 (46%)	24 (32%)	1 (1%)	10 (14%)	5 (7%)
**Diabetes prevention** **programme**	28 (38%)	35 (47%)	18 (24%)	1 (1%)	14 (19%)	2 (3%)
**Psychological support**	24 (32%)	26 (35%)	12 (16%)	0	28 (38%)	5 (7%)
**Social prescribing**	22 (30%)	28 (38%)	25 (34%)	0	23 (31%)	4 (5%)

In 12 LAs, respondents reported that referral processes had changed following the Covid-19 pause. Changes were mainly positive, with increased accessibility to self-referrals and clearer gateways to a range of services.

### Monitoring and evaluation

The number of LAs that monitored the outcomes of Health Check programmes was relatively low. Twenty-two (31%) respondents gathered data on GP referrals, and 30 (42%) on the outcomes from such referrals, e.g., new diagnoses; 24 (34%) gathered data on referrals to lifestyle support services, and 19 (26%) on the outcomes from these, e.g., weight loss. Information gathered on the uptake of prescriptions (8; 11%), and on the outcomes of prescribing, e.g., lowered cholesterol (4; 6%) was even lower. Fewer than half the LAs (33; 46%) reported any evaluation or assessment of their local programme in the last five years.

### Associations between delivery category and other variables

An association was found between the delivery category and the total number of commissioned follow-on services reported. The mean number of commissioned services was significantly higher in the LAs categorised ‘blended with outreach’ (7.6) compared to ‘general practice’ (6.6) or ‘blended’ (5.9) (F = 3.85; p = 0.026). The LAs falling in the blended with outreach delivery category also had the highest mean number of referral processes (8.0), compared to those categorised as general practice (7.5) and blended delivery (7.0), although the difference was not statistically significant (F = 0.311, p = 0.764).

Respondents’ confidence in the capacity, accessibility and usage of the support services varied little across the categories of delivery, although trends were visible in the confidence in accessibility and usage with the increasing use of non-general practice-based delivery ( Table 6).

**Table 6.** Reported confidence levels in capacity, accessibility and usage of support services by delivery category (where n is number of respondents).

Delivery category	Overall confidence ratings for all services (where 1 means ‘not confident at all’ and 4 means ‘very confident’)		
	Capacity average (n = 66)	Accessibility average (n = 67)	Usage (single rating) (n = 68)
**General practice**	**2.76 ± 0.67**	**2.68 ± 0.67**	**2.00 ± 0.73**
**Blended**	**2.62 ± 0.80**	**2.74 ± 0.75**	**2.10 ± 0.70**
**Blended with outreach**	**2.74 ± 0.79**	**2.77 ± 0.71**	**2.48 ± 0.89**

The number of methods used to prioritise invitations was not related to the category of delivery, but LAs in the blended and blended with outreach categories reported a greater increase in the number of factors used post-Covid pause compared to LAs in the general practice delivery category. The degree of monitoring and evaluation reported as being undertaken was also unrelated to the category of delivery.

**Figure 4.**
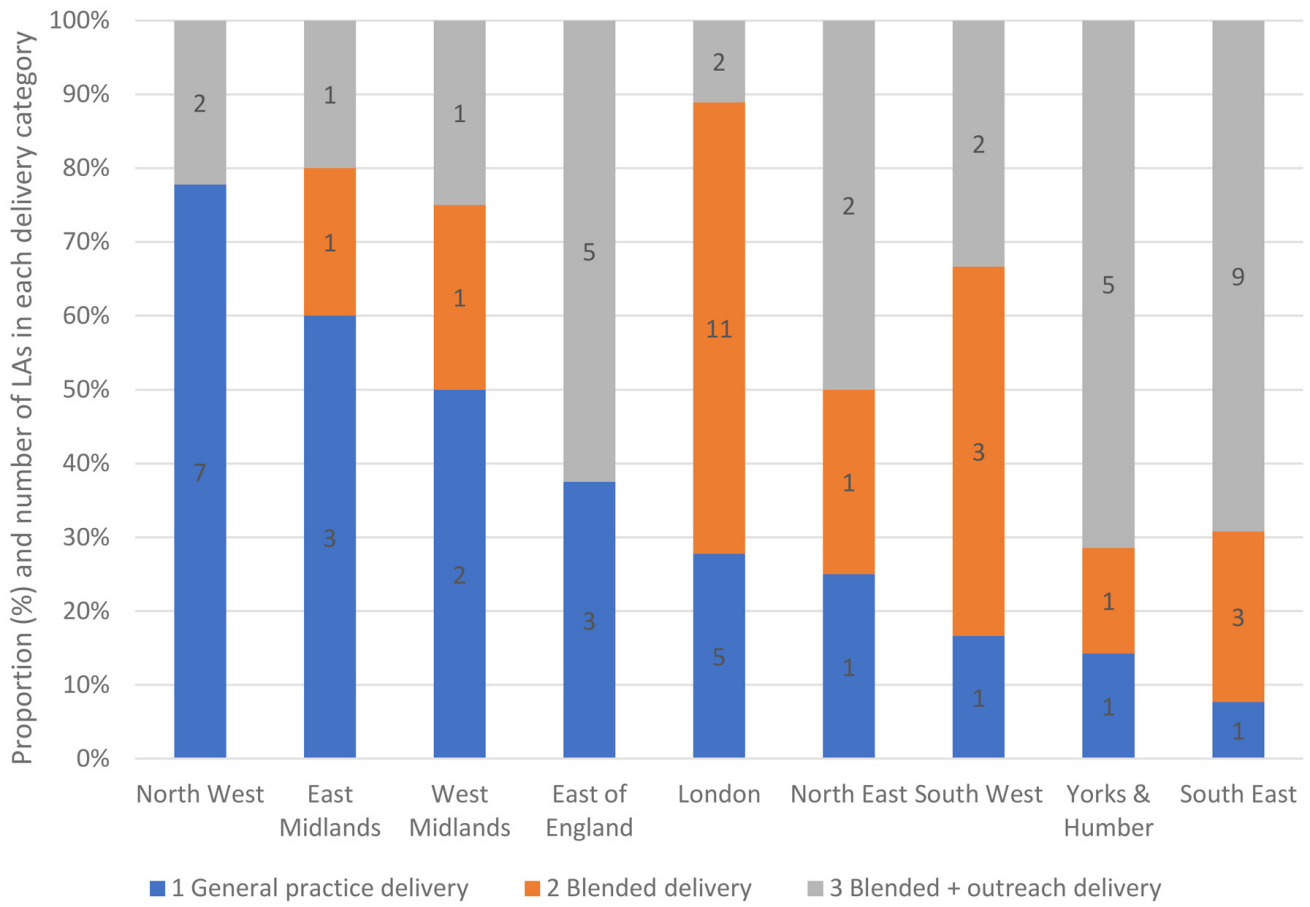
Proportion of LAs in the Health Check delivery categories by geographic region.

There were notable differences in the proportions of LAs in the three delivery categories in each geographical region. Whilst five out of the seven responding LAs in Yorkshire and Humber fell in the blended with outreach category, seven out of the nine responding LAs in the North West fell in the general practice delivery category ( Figure 4).

There were no significant differences in the relative size of the LAs falling into each delivery category, although the LAs delivering via community outreach tended to be larger than those in other categories.

**Figure 5.**
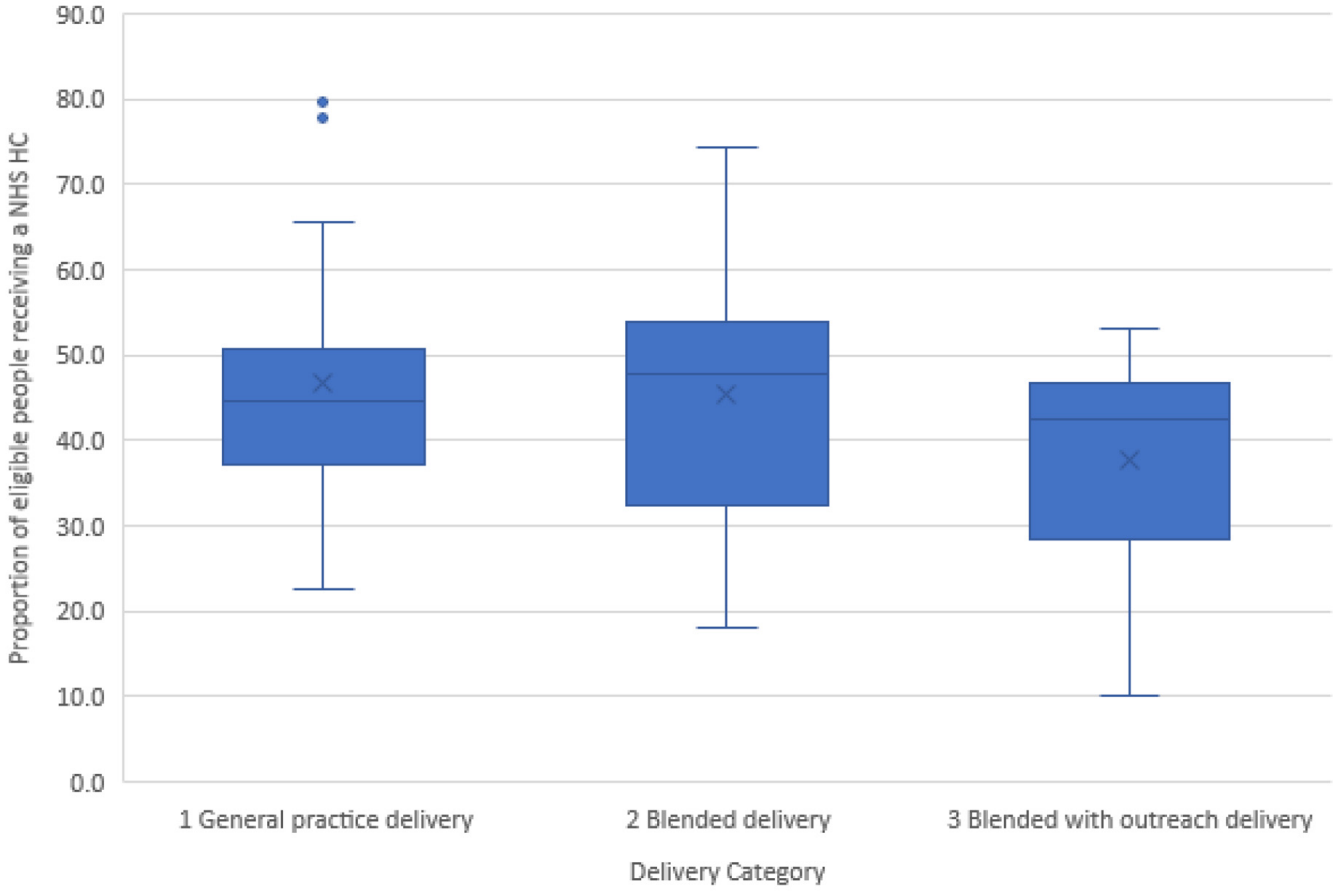
Health Check delivery performance by category of delivery.

There were also no significant differences in the relative size of the public health budgets of LAs, or in the relative deprivation of LAs falling in each delivery category. With regards to Health Check programme delivery performance, LAs delivering Checks using a blended with outreach model achieved, on average, a lower coverage (37.8%) than those delivering using a general practice (46.7%) or blended (45.4%) delivery model (F = 3.217; p = 0.046). However, as the boxplots show, the medians (the lines within the boxes), interquartile ranges (the box lengths) and the overall spread (whiskers) were overlapping, indicating that the categories were not significantly different in respect to programme delivery performance ( Figure 5).

## Discussion

This is the first national survey of commissioning of the NHS Health Check programme which was not undertaken by PHE, with a sample that includes half of all LAs and a spread of authorities across the nine regions. It is also the first to focus on the support given to Health Check attendees to make lifestyle changes following the risk assessment, and to attempt to categorise methods of delivery, in consultation with a professional stakeholder group. The timing of the survey is also important; it clearly demonstrates the variation in delivery of the Health Check before the Covid pause in terms of where and how it was provided and by whom, and some of the innovations in delivery that have emerged since. The variation and innovation in delivery found in the survey resonated with members of our professional and PPI groups, who further illustrated the findings by describing multiple examples from their own experience. Input from these groups helped us to confirm and interpret our survey findings. Yet, the study has a number of limitations. The possibility of sampling bias or social desirability cannot be ruled out. Our data suggest that amongst those who responded, there may have been potential bias towards enthusiastic commissioners who are champions of the programme. Our response rate was relatively low in comparison with the previous national surveys, which is unsurprising given the timing of the survey during the pandemic. There are, therefore, still many gaps in the national picture. Moreover, there were questions (for instance relating to availability of and referral routes for some follow-on services) about which some commissioners might not have had the relevant knowledge. A national survey of Health Check providers would be a valuable complement to the commissioner survey.

Two previous national surveys reported which provider organisations deliver the Health Check, but not which health professionals ^15, 16^. We found that whilst healthcare assistants and/or nurses delivered the Check in most responding LAs, GPs delivered at least some of the Checks in two thirds of the LAs. A wide range of other professionals delivered the Checks to differing extents in some LAs, although some commissioners were not aware of who delivered them. Attention to *who* is providing the Checks on the ground is important given the variation in skills, experience and confidence amongst different professionals, and the extent to which they are likely to face challenges related to workload, information technology, funding and training ^19– 21^. These issues are all likely to influence patient experiences and outcomes ^20, 22^. 

Our findings confirm that most LAs commission at least one service to support key aspects of behavioural change. However, there are some LAs in which significant service gaps exist. This is consistent with previous research demonstrating gaps in weight management services ^23^, and the reductions in spending by LAs on a range of support services including smoking cessation ^24^. It was notable, given the importance of obesity as a CVD risk factor ^25^, that four of the LAs reported no weight management services being commissioned at all. Given the frequency with which LAs review and change public health service commissioning ^26^, it is useful to update data from the PHE survey in 2014, which found that the majority of LAs provided lifestyle interventions including stop smoking services (96%), exercise referral (89%), weight management (84%) and diabetes prevention (58%). Amongst our sample, only five (5%) mentioned exercise referral in the ‘any other services’ comment box. However, this might be underestimated since they could have been included as part of either weight management or diabetes prevention services. Following guidance from our professional stakeholder group, we did not include exercise referral schemes in the list of services in the survey question. We also found signs of increasing fragmentation and complexity in commissioning since the 2014 survey, with support services being delivered by a range of different public, private and third sector providers, often with a mix in each area.

The clear variation in availability and accessibility of support services, together with the diverse ways in which the programme is delivered, and the complexity of the commissioning landscape in which interdependent services are commissioned and provided by a range of different organisations, is likely confusing for both Health Check providers and attendees. Whilst most commissioners feel reasonably confident about the capacity and accessibility of support services to accept referrals for Health Check attendees, they are much less confident that appropriate use is being made of them by Health Check providers. This points to a disconnect between the intended aim of the programme, where the intention is to follow through risk communication with appropriate support, and the way the programme is delivered in many areas, where providers are not necessarily linking (or able to link) attendees to appropriate support. Respondents were least certain about whether psychological support services had sufficient capacity and accessibility, and had least awareness of the referral processes for them. This may be because these services are generally commissioned by NHS organisations, so LA commissioners know less about them. However, whilst psychological services are less central to the risk management aim of the programme, the Health Check is sometimes a potential conduit to them, and they are a relevant adjunct to lifestyle support services. They play an important part in the assessment and treatment of a range of motivational, behavioural, and cognitive/affective factors implicated in overweight, for example ^27^.

Various changes in programme delivery appear to have been prompted by the pandemic. Delivery had yet to resume in ten of our responding LAs. In those that had resumed, the use of community venues had decreased, and the use of alternative (remote) methods of delivery had increased. The shifts in location and the demands of the pandemic response meant some changes were seen in who delivered the Checks. Some commissioners also appear to be placing a greater emphasis on prioritising specific groups of eligible candidates for a Health Check following the pandemic. In relation to the capacity and accessibility of support services for Health Check attendees, our data points to both challenges, for example with services being overwhelmed, and opportunities, such as greater investment in specific areas. The moving of support services to online provision was described as both a challenge and an opportunity. These changes will need careful evaluation in relation to issues such as digital exclusion ^28^, impacts on inequalities, and the quality, efficiency and cost-effectiveness of Health Check delivery.

Of previous surveys we have identified, only one other ^29^ sought information on programme monitoring. This reported that three of the eight commissioners sampled gathered data on advice offered, four on the number of referrals and four on outcomes, such as new diagnoses and new statin prescriptions. These authors considered that such monitoring was an important ingredient of the programme’s effectiveness. Our survey suggests that little has changed, with only 41% gathering data on outcomes of referrals to GPs. There is evidence to suggest that the lack of emphasis placed on monitoring and follow-up is something that Health Check attendees also find frustrating ^30^. Moreover, the lack of a requirement to gather such information for the national dataset and the omission of this topic in the previous two national surveys suggest that outcomes data will continue to be sparse for the foreseeable future.

The novel typology of delivery modes, based on our survey data, identified three main models in use prior to the Covid-19 pause. We found more LAs reportedly commissioning pharmacy or outreach methods compared with the 2020 national survey ^16^. In addition, the 2020 survey found that 57% *only* delivered the Health Check in general practice, compared to 32% in our survey. We found only two other surveys of commissioners, both small in scope: a survey of eight London Primary Care Trusts (PCTs) conducted in 2010 ^29^ and a survey of 16 LAs achieving high uptake rates conducted in 2019 ^31^. Two of the eight PCTs surveyed in 2010 used models akin to blended with outreach, while six of those surveyed in 2019 had some form of outreach, which included pharmacy provision. There appeared to be regional differences in the commissioning of delivery models, with a high proportion of general practice only delivery in the North West, while LAs offering blended with outreach delivery tended to have larger populations. It is not clear what is behind this regional variation in models of provision.

LAs using a blended with outreach model tend to have a higher number of support services to support behaviour change following the check and greater confidence in the accessibility and usage of these services, although there was no association between delivery model and public health budgets. The blended with outreach model is also associated with more post-Covid-19 prioritisation of Health Check candidates. However, the evidence did not suggest that the use of this model significantly influenced coverage, in terms of the proportion of eligible people receiving the Check. This might point to the fallibility of using overall coverage as an indicator of best practice where a more targeted approach, using a range of methods to reach those who might benefit the most, might deliver the greatest gains for population health, particularly in terms of addressing health inequalities. However, given the general assumption that improving access (e.g., through outreach provision) will encourage more people to take up the offer of a Check ^32^, the further examination of delivery models in relation to outcomes is warranted. A comparison of delivery models according to outcomes other than programme coverage would also be valuable, particularly in the context of health inequalities and the Government’s ‘Levelling Up’ agenda ^3^. However, this would require the gaps in reporting of key outcomes data to be addressed.

## Conclusions

This study has confirmed the variation in delivery of the Health Check programme identified in previous surveys and has provided further insight into the ways in which delivery is continuing to change – particularly in response to the Covid-19 pandemic - both in relation to who delivers the check, and how it is delivered. Our findings also highlight the gaps and variation in support services that Health Check attendees might be referred to, and which are so important for achieving programme impact. 

Our findings support the description, for the first time, of a basic typology of NHS Health Check programme delivery. However, given the lack of association between delivery models and programme uptake/coverage, we cannot advocate any particular model. There is a real need for more monitoring, to enable comparisons to be made concerning important outcomes such as lifestyle changes. Similarly, the lack of any association with public health budget suggests that it is not the amount of money available which is important, but how it is spent (i.e., what services are commissioned and provided). The pandemic may have provided an impetus to identify highest risk individuals, which might encourage more LAs to prioritise invitations according to specific risk factors, particularly as they deal with the inevitable backlog resulting from the pause in delivery. This could have implications for programme impact, particularly in reducing health inequalities, but there is a need to ensure that risk assessments are followed up with appropriate support, and that services for onward referral exist and are used appropriately by Health Check providers. Our study suggests there is some way to go in achieving this. 

## Data Availability

Open Science Framework: Underlying data for ‘The NHS Health Check programme: a survey of programme delivery in England before and after the Covid-19 pandemic response’, https://www.doi.org/10.17605/OSF.IO/N84JV ^18^ This project contains the following underlying data: Responses for 74 LAs suitable for data sharing 280323.xlsx Some responses to open-ended questions were removed from the shared dataset to preserve the anonymity of the responding organisation. Readers/reviewers wishing to apply for access to the full dataset (for academic non-commercial research purposes) should contact Dr Erica Gadsby e.j.gadsby@stir.ac.uk. Data will be shared on the condition that ethical requirements are complied with and the privacy of research participants can be safeguarded. Open Science Framework: Extended data for ‘The NHS Health Check programme: a survey of programme delivery in England before and after the Covid-19 pandemic response’, https://www.doi.org/10.17605/OSF.IO/N84JV ^18^ This project contains the following extended data: NHS Health Check programme: Introduction and description of files.docx Survey post pilot.pdf Data are available under the terms of the Creative Commons Zero “No rights reserved” data waiver (CC0 1.0 Public domain dedication).
