# Supplementary material for: The NHS Health Check programme: a survey of programme delivery in England before and after the Covid-19 pandemic response
Source: NIHR Open Res. 2023 Aug 25;3:32. Originally published 2023 Jun 22. [Version 2] doi: 10.3310/nihropenres.13436.2 (PMC10593320; doi:10.3310/nihropenres.13436.2)
Supplement: Peer review history

# Reviewer report: Reviewer response for version 2

**DOI:** 10.3310/nihropenres.14617.r30321

**Published:** 2023-09-08

**Authors:** Maria Guzman-Castillo

**Affiliations:** 1 Department of Social Research, University of Helsinki, Helsinki, Finland

I approved the revision. The authors did not incorporate all my comments but those were mainly to improve readability of the text.

Is the work clearly and accurately presented and does it cite the current literature?

Yes

If applicable, is the statistical analysis and its interpretation appropriate?

Yes

Are all the source data underlying the results available to ensure full reproducibility?

No source data required

Is the study design appropriate and is the work technically sound?

Yes

Are the conclusions drawn adequately supported by the results?

Yes

Are sufficient details of methods and analysis provided to allow replication by others?

Partly

Reviewer Expertise:

NA

I confirm that I have read this submission and believe that I have an appropriate level of expertise to confirm that it is of an acceptable scientific standard.

# Reviewer report: Reviewer response for version 1

**DOI:** 10.3310/nihropenres.14574.r29604

**Published:** 2023-08-03

**Authors:** Carissa Bonner

**Affiliations:** 1 Faculty of Medicine and Health, School of Public Health, The University of Sydney, Sydney, New South Wales, Australia

This is a well written report of a survey examining NHS health check delivery and changes during COVID-19, with stakeholder input to inform the design. I have minor comments that could be considered to improve the clarity of the paper.

**Introduction sections**

 Abstract – conclusion doesn’t quite follow from results (innovation and commissioning not mentioned); add base rate for % response (i.e. 151 areas)

 Lay summary – some parts are complex especially last paragraph, aim for grade 8 and explain complex phrases like public health commissioners

 Introduction gives a clear overview of the context

**Methods**

 Methods is detailed and mostly clear

 Can you clarify how much data was “amended” in response to open comments?

 How was the typology developed? In abstract it sounds like it was derived from the data but here it sounds like it was a priori

**Results**

 Here you say you remove duplicates but in methods you say you used duplicates for individuals answering for multiple regions – can you clarify how may duplicates there were and how you identified them as needing removal

 Can you give some context for how representative your survey is by region

 Table 3/4/5 – add percentages throughout results text and tables; this is reported in some sections but not others so should be consistent

 Figures are clear

 Table 7 – report SD with mean

**Discussion**

 Good comparison to other relevant surveys

 Novel typology is emphasised so important to explain how this was derived a bit more

Is the work clearly and accurately presented and does it cite the current literature?

Yes

If applicable, is the statistical analysis and its interpretation appropriate?

Yes

Are all the source data underlying the results available to ensure full reproducibility?

Yes

Is the study design appropriate and is the work technically sound?

Yes

Are the conclusions drawn adequately supported by the results?

Yes

Are sufficient details of methods and analysis provided to allow replication by others?

Yes

Reviewer Expertise:

CVD risk assessment, implementation, general practice, risk communication

I confirm that I have read this submission and believe that I have an appropriate level of expertise to confirm that it is of an acceptable scientific standard.

# Response

**Published:** 2023-08-18

**Authors:** Erica Gadsby

**Affiliations:** Health Sciences and Sport, University of Stirling, Stirling, Scotland, UK

We would like to thank the reviewer for their careful reading, positive feedback and helpful comments. In response to the points raised:

 1. Abstract - The ‘key innovations’ mentioned in the conclusions related to the ‘continued changes’ mentioned in the results. This has been clarified by changing the wording to ‘continued changes’ in both sections. The conclusion section now refers to ‘commissioners’ intentions’. We have also added (of 151) after number of responding LAs to clarify base rate.

 2. Lay summary - Thank you for this feedback. We have revised the lay summary. The Hemingway App now judges it as ‘Good’ readability at Grade 8 level.

 3. Methods - Thank you for this point. We have further added “… where the open comments sometimes provided further clarification or detail. One response was amended, where the respondent had not ticked ‘pharmacist’ as a provider, but had told us in a free text comment that pharmacists were used to deliver the Checks.”

 4. Results

- We have now clarified that the typology was ‘ *derived from the data, in consultation with our stakeholder group*’ in the data analysis section. We have removed Table 1 from the methods section to help make this clearer, and renumbered subsequent tables.
- We have now clarified in the text: There were four LAs for which there were two identical responses. Removal of these duplicates left 68 responses …
- The representativeness by region is illustrated in table 2: survey respondents by region.
- Percentages have now been added to results tables. Thank you for your comment that the figures are clear.
- Table 7 now reports SD with mean
- We have added the following to the results section: “ *A typology of delivery was developed through an iterative process, looking across the data to compare and contrast delivery locations, modes and staff involved (these aspects are presented in more detail in the sub-sections below). The professional stakeholder group were consulted regarding various iterations of the typology.*”

# Reviewer report: Reviewer response for version 1

**DOI:** 10.3310/nihropenres.14574.r29610

**Published:** 2023-08-03

**Authors:** Maria Guzman-Castillo

**Affiliations:** 1 Department of Social Research, University of Helsinki, Helsinki, Finland

I congratulate the authors on an exceptionally well-written manuscript. I have provided some additional comments for your consideration:

1. The results section should incorporate the involvement of the public and stakeholder engagement meetings discussed in the methods, to show how their views influenced the results. Currently, the results only include the analysis of the survey.
2. It would be beneficial to include uptake statistics for each region in Table 2.
3. Tables 4-7 should include the percentage (%) values, and it would be helpful to remind the reader of the total number of LAs.
4. The term "delivery" has two different meanings in the manuscript, which makes it difficult to follow the authors' intent. The first meaning refers to the physical place, as discussed in "Delivery of the NHS Health Check." However, in the section on "Associations between delivery category and other variables," the authors use "delivery" to refer to the "typology of delivery," which encompasses not only the physical place but also the health professionals involved.
5. The authors could reduce the text on "Delivery of the NHS Health Check" and "Health professionals involved in Health Check delivery" and instead provide more details on the "typology of delivery." These three sections currently overlap, making them sound repetitive and unnecessary. Additionally, considering moving tables 2 and 3 to the appendix and grouping these sections together to improve the flow of the paper.
6. Adding the percentage (%) values within each of the color bars in figures 1-3 would facilitate the reader in calculating the exact percentages more easily.
7. Provide keys or explanations to help readers interpret the boxplot in figure 4. It is important to consider that some readers may not be familiar with how to interpret whiskers.
8. In figure 4, the whiskers and boxes overlap for the three categories, which indicates that we cannot state the groups are different.
9. Ensure consistency when discussing changes pre/post-Covid. Some sections discuss changes in the table, while others discuss them in the text. Create a new subsection specifically dedicated to discussing changes after Covid to maintain consistency throughout the manuscript.
10. Since the typology of delivery seems to be one of the main elements of the paper, consider using a data visualization tool to effectively represent it.
11. It would be beneficial to add some key results at the beginning of the discussion section.
12. Discuss the public health implications of this study more comprehensively. Currently, some ideas are scattered throughout the manuscript, so it would be better to consolidate them into a single paragraph.
13. The discussion focuses heavily on the differences in coverage among different typologies of delivery, but as previously stated, the boxplot indicates that there are no differences between the groups. This should be acknowledged and addressed in the discussion.

Is the work clearly and accurately presented and does it cite the current literature?

Yes

If applicable, is the statistical analysis and its interpretation appropriate?

Yes

Are all the source data underlying the results available to ensure full reproducibility?

No source data required

Is the study design appropriate and is the work technically sound?

Yes

Are the conclusions drawn adequately supported by the results?

Yes

Are sufficient details of methods and analysis provided to allow replication by others?

Partly

Reviewer Expertise:

Public Health modelling

I confirm that I have read this submission and believe that I have an appropriate level of expertise to confirm that it is of an acceptable scientific standard, however I have significant reservations, as outlined above.

# Response

**Published:** 2023-08-18

**Authors:** Erica Gadsby

**Affiliations:** Health Sciences and Sport, University of Stirling, Stirling, Scotland, UK

We would like to thank the reviewer for their careful reading, positive feedback and helpful suggestions. Below, we response to each point in turn:

 1. The contributions from public and stakeholder contributors were not treated as research data, and so are not incorporated in the results section. However, we have included some additional text in the discussion section to highlight the ways in which these contributions helped to shape our analysis and interpretation of the research findings.

 2. Thank you for this point. We think you mean the NHS Health Check performance figures (as in uptake of checks)? The authors discussed this at length and decided it wouldn’t be useful to include these statistics in table 2. Table 2 is reporting the representativeness of the survey by region (in terms of how many and what proportion of LAs in each region responded to the survey). We reasoned that including a snapshot of NHS Health Check performance data within this table would be confusing.

 3. Total number of LAs and percentages for each cell have now been added to tables 4-6. Table 7 presents average confidence scores of respondents.

 4. Thank you for this feedback. The section titled ‘Delivery of the NHS Health Check’ discusses both location (i.e. physical place) and mode of delivery (e.g. remote). The typology of delivery does include information on health professionals as well. To make terminology clearer throughout, we have moved the sub-section ‘Typology of NHS Health Check delivery’ to precede the detail on location, mode and professionals involved (see point 5). We have also changed the subheading ‘Delivery of the NHS Health Check’ to ‘Location and mode of Health Check delivery’.

 5. Thank you for this point. We have moved the section ‘Typology of local authorities’ (and re-named it ‘Typology of NHS Health Check delivery’) up to the start of the results section. This helpfully precedes the further detail on location, mode and personnel in the subsequent sections. Having done this, we felt (former) Table 1 was no longer required so we have deleted this and renumbered subsequent tables. We felt that given the importance of regional coverage and detail related to health professionals involved, we would prefer to keep these in the main body of the article.

 6. Given the number of colour bars in figures 1 and 2 we felt that adding percentage values made them less clear. Since Reviewer 2 commented that the figures are clear, we have opted not to add them. However, we have chosen to add values (as opposed to percentages) in the colour bars in figure 3, since we feel this is quite helpful. We have added a title to the vertical axis to make this clear.

 7. We have amended the wording preceding the boxplots to help readers interpret them.

 8. In the amended wording preceding the boxplots we have included a clarification of our interpretation.

 9. All sections in the results discuss pre/post covid in the text except ‘monitoring and evaluation’, where it is not relevant to do so. We have included an additional sentence and slightly changed the ordering of text in the section ‘health professionals involved in Health Check delivery’ to better highlight changes post Covid. We felt it would lead to too much repetition to have a separate section in results that deals just with changes after Covid. There is a paragraph in the discussion section that is specifically dedicated to discussing changes after Covid. Given this was quite brief, we have added some additional text here.

 10. We have included a proposed Figure 1. We hope this is along the lines that Review 1 was thinking.

 11. Given the range of results presented, we felt this would lead to too much repetition. We have checked that the Discussion section summarises and discusses each sub-section of results in turn.

 12. The public health implications of this study are dealt with in the Discussion section where we summarise each key aspect of the results and the public health implications in turn. First, we discuss the findings related to which professionals provide the checks, and highlight the implications in terms of patient experiences and outcomes of the Checks. Next, we discuss findings related to follow-on support services, and highlight the implications in terms of risk reduction/management and inequalities in access to services. Next, we discuss findings related to changes in programme delivery prompted by the pandemic, and the implications in terms of access to services, prioritisation, digital exclusion, inequalities, and service quality, efficiency and cost-effectiveness. Next, we discuss findings related to monitoring and evaluation, and the implications in terms of our ability to evaluate the impact of the programme. Finally, we link this lack of monitoring and evaluation data to the diversity in delivery models, and highlight how little we know about how best to deliver the programme (particularly to reduce health inequalities). We believe this is a more thorough approach than trying to deal with all public health implications in one paragraph. The final paragraph of the conclusion section further summarises these implications.

 13. In the paragraph that discusses associations between delivery category and other variables, we have changed one sentence to clarify the lack of association between delivery category and programme performance.
